# Mesenchymal stem and non-stem cell surgery, rescue, and regeneration in glaucomatous optic neuropathy

**DOI:** 10.1186/s13287-021-02351-4

**Published:** 2021-05-06

**Authors:** Paolo Giuseppe Limoli, Celeste Limoli, Enzo Maria Vingolo, Federica Franzone, Marcella Nebbioso

**Affiliations:** 1 Low Vision Research Centre of Milan, Piazza Sempione 3, 20145 Milan, Italy; 2 Department of Sense Organs, Faculty of Medicine and Odontology, Sapienza University of Rome, P.le A. Moro 5, 00185 Rome, Italy; 3 Department of Sense Organs, Ocular Electrophysiology Centre, Umberto I Policlinic, Sapienza University of Rome, Viale del Policlinico 155, 00161 Rome, Italy

**Keywords:** Adipose-derived stem cell, Autograft, Cell surgery, Glaucoma, Glaucomatous optic neuropathy, Growth factor (GF), Limoli retinal restoration technique (LRRT), Mesenchymal stem cell (MSC), Platelet-rich plasma, Stem cells

## Abstract

**Background:**

Glaucomatous optic neuropathy (GON) is an anatomofunctional impairment of the optic nerve triggered by glaucoma. Recently, growth factors (GFs) have been shown to produce retinal neuroenhancement. The suprachoroidal autograft of mesenchymal stem cells (MSCs) by the Limoli retinal restoration technique (LRRT) has proven to achieve retinal neuroenhancement by producing GF directly into the choroidal space. This retrospectively registered clinical study investigated the visual function changes in patients with GON treated with LRRT.

**Methods:**

Twenty-five patients (35 eyes) with GON in progressive disease conditions were included in the study. Each patient underwent a comprehensive ocular examination, including the analysis of best corrected visual acuity (BCVA) for far and near visus, sensitivity by Maia microperimetry, and the study of the spectral domain-optical coherence tomography (SD-OCT). The patients were divided into two groups: a control group, consisting of 21 eyes (average age 72.2 years, range 50–83), and an LRRT group, consisting of 14 eyes (average age 67.4, range 50–84).

**Results:**

After 6 months, the BCVA, close-up visus, and microperimetric sensitivity significantly improved in the LRRT-treated group (*p*<0.05), whereas the mean increases were not statistically significant in controls (*p*>0.5).

**Conclusions:**

Patients with GON treated with LRRT showed a significant increase in visual performance (VP) both in BCVA and sensitivity and an improvement of residual close-up visus, in the comparison between the LRRT results and the control group. Further studies will be needed to establish the actual significance of the reported findings.

## Background

Glaucoma causes retinal ganglion cell (RGC) layer degeneration, impairing visual function. It has been estimated that over 100 million people will be affected by glaucoma in 2040 [1]. Glaucoma affects the sensory input towards the visual cortex resulting in progressive visual field loss [2]. Anatomical degeneration can be found since the early stages of glaucoma also within the lateral geniculate nucleus and visual cortex [2]. The critical intraocular pressure (IOP), representing chronic stress, is considered the main cause of damage to the RGC layer and nerve fibers. IOP has been demonstrated to reduce bidirectional axonal transport over time, according to both individual susceptibility and clinical presentation [3]. The damage seems to initially involve the unmyelinated RGC axons within the optic nerve head and then the RGC bodies, resulting in glaucomatous optic neuropathy (GON). In that degenerative phase, thinning of the neuroretinal rim and pathological cupping have been observed, resulting in specific nervous fiber damage corresponding to visual field defects.

Numerous pathogenetic mechanisms have been proposed to explain the apoptotic loss of RGCs in glaucoma: microcirculatory ischemia, hyperproduction of reactive oxygen species (ROS), impaired bidirectional axonal transport, parainflammation, excitotoxicity, neurotrophic factors reduction, and electrical activity reduction [2–5]. Currently, the most frequently used treatment for GON is IOP control, being considered the main risk factor [3]. Hypotensive therapies, together with neurotrophic supplements, represent the recommended treatment for patients with GON to stop or slow down neurodegeneration.

The recent appearance of cell therapy in regenerative medicine has represented a promising tool in glaucoma therapy [6]. On the one hand, embryonic stem cells (ESC) and induced pluripotent stem cells (iPSC) have been used in preclinical and clinical studies to replace dead or diseased RGCs, but, although expressing RGC markers, they have not shown to effectively restore retinal connections, as they remain close to the injection site [6].

On the other hand, mesenchymal stem cells (MSCs) can be exploited for their paracrine secretion of different molecules that have been shown to activate RGC-intrinsic regenerative programs after optic nerve injury, promoting cell survival and axonal regeneration [7]. Specifically, the effectiveness of MSCs is expressed through several mechanisms, including hemorheological, anti-oxidative, anti-inflammatory, anti-apoptotic, neurotrophic, and cytoprotective ones [3, 6, 7]. These mechanisms clinically could lead to the improvement of visual performance (VP) and the overall prognosis of glaucoma. In fact, the majority of our patients are affected by low vision, seeking an improvement of their visual performance. This premise clarifies the choice of the evaluate indicators. The latter one can be achieved not only through the aid of magnifying devices but also through neuromodulation and neuroenhancement techniques.

**Fig. 1:**
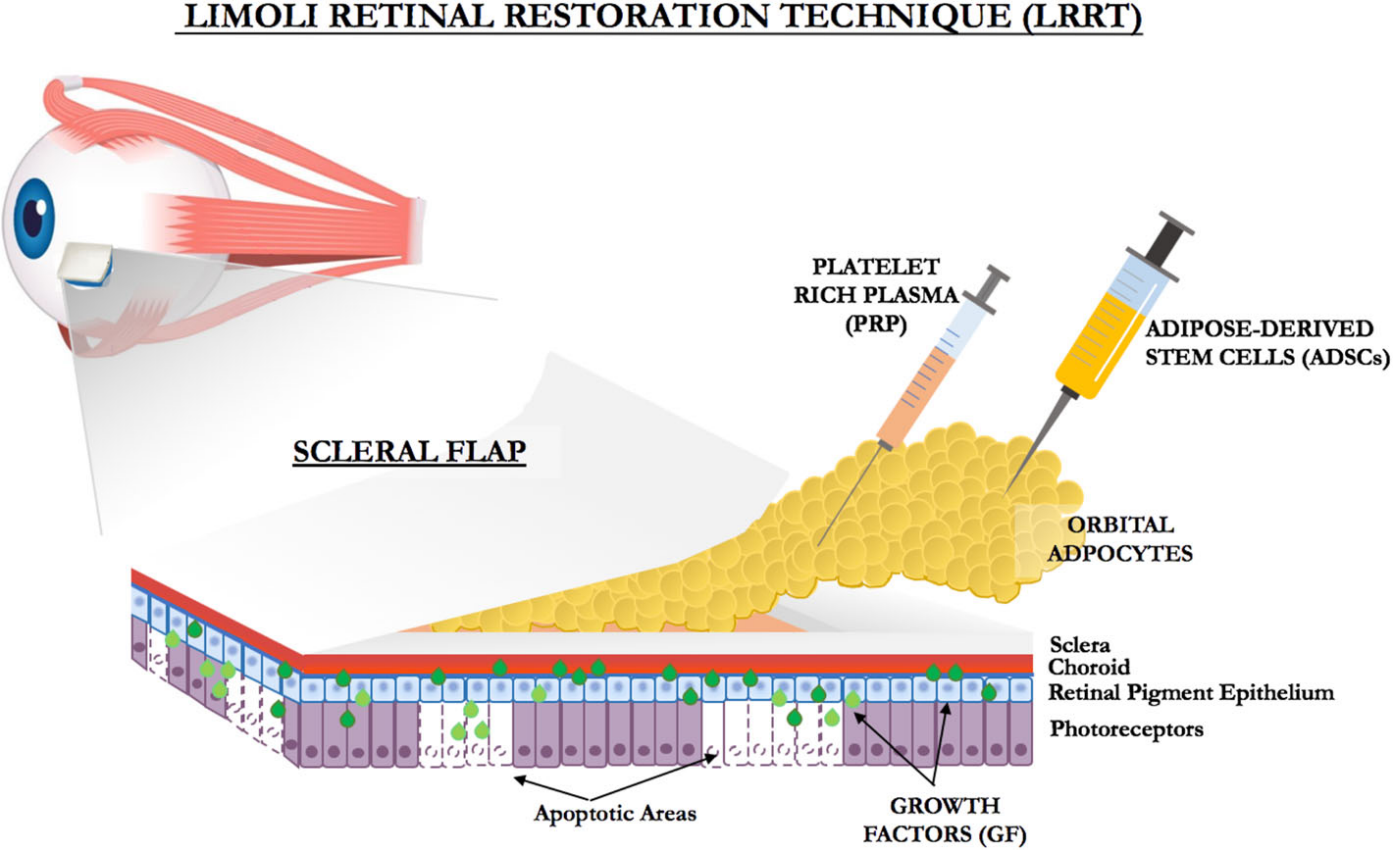
Representation of Limoli retinal restoration technique (LRRT) suprachoroidal autologous mesenchymal stem cells (MSC) graft, platelet-rich plasma (PRP), ADSCs, and growth factors (GF)

In this context, an autograft of MSCs in the suprachoroidal space according to the Limoli retinal restoration technique (LRRT), consisting of the following triad, could be effective: adipose stromal cells (ASCs), adipose-derived stem cells (ADSCs) contained in the stromal vascular fraction (SVF) of adipose tissue, and platelets (PLTs) recovered from the platelet-rich plasma (PRP) [8–13] (Fig. 1).

This study aimed to evaluate both the efficacy and safety of LRRT suprachoroidal MSC graft in patients with GON.

## Materials and methods

### Study participants

This retrospectively registered study was performed at the Low Vision Center in Milan between January 2015 and September 2019. The study was performed in accordance with the tenets of the 1964 Declaration of Helsinki and was approved by the Institutional Review Board of the Low Vision Academy (No. 2014/MC104, 4 October 2014). Written informed consent was obtained from all the patients prior to enrollment.

Furthermore, patients with minor damage preferred clinical follow-up without surgery because they did not feel the damage progression, whereas treated patients chose surgery as they felt that damage progression, despite the good tonometric control. Many patients included in the study were pseudophakic, and the ones still phakic did not have a significant cataract that could interfere with vision. Individuals who met the following inclusion criteria were recruited for the study.

Diagnosis of GON highlighted by microperimetry or, when necessary, coherence optical tomography (OCT):

- Excavated optic papilla with cup/disc <0.6.
- Good therapeutic balance (IOP ≤15 mmHg) obtained with hypotonizing therapy; the pressure must be corrected also after LRRT surgery.
- Best corrected visual acuity (BCVA) between +1 and 0 logarithm of the minimum angle of resolution (LogMAR).
- Age between 50 and 84 years old.
- Stable eye conditions without previous surgery for ocular trauma.

Subjects who met any of the following criteria were excluded from the study:

- Bad therapeutic balance (IOP > 15 mmHg) obtained with hypotonizing therapy; IOP must be also corrected after LRRT surgery.
- Refractive error ≥ 6 diopters of myopia, hypermetropia, and astigmatism.
- Close-up visus not evaluable.
- Presence of cataract or other media opacity that could interfere with a functional response.
- Presence of chorioretinal diseases including macular pucker with the altered foveal area, age-related macular diseases (AMD), or eredodistrophy.
- Intravitreal injection treatment and/or intraocular surgery.
- Inability to provide written informed consent.
- Inability to attend all follow-up visits.
- Systemic diseases including multiple sclerosis, epilepsy, vasculitis, Parkinson’s disease, renal and hepatic diseases, malignant neoplasms, and decompensated diabetes mellitus.

Some patients presented indications for bilateral treatment (both eyes were visually impaired), and other cases presented indications for unilateral treatment (contralateral blind or normal or only slightly visually impaired). Or according to the patient’s consent.

All the eyes enrolled in this study were divided into two groups: the LRRT group who underwent autologous suprachoroidal graft of mesenchymal cells and the control group of GON patients who did not undergo LRRT surgery. Participants used as control were matched with GON patients according to the sensitivity alteration measured by microperimetry.

### Ophthalmologic examination

The diagnosis of GON was established for each patient by the clinical analysis of visual performances (VP). Then, evaluation by slit-lamp biomicroscopy with and without dilatation, applanation tonometry, and retinal mapping with an indirect ophthalmoscope was performed. Also, BCVA, close-up visus, sensitivity measured by microperimetry (MY) with Maia 100809 (CenterVue S.p.A., Padua, Italy), spectral domain-optical coherence tomography (SD-OCT) with Cirrus 5000 (Carl Zeiss Meditec AG, Jena, Germany), and ocular electrophysiology with the Retimax electromedical system (C.S.O. Srl, Scandicci, Italy) were performed. All the ophthalmologic analyses were carried out by the same examiner at baseline (T0) and 6 months (T180) in both groups. Finally, the subjective improvement of VP in the LRRT group at 6 months after surgery was reported. BCVA was always measured according to the standards recommended by the early treatment diabetic retinopathy study charts (ETDRS) at 4 m and expressed in logMAR. The visual acuity for near distance (close-up visus) was recorded in points (pts). Microperimetry was performed using a Maia apparatus (Centervue spa, Padua, Italy) with images acquired by scanning laser ophthalmoscopy. The perimetry can indicate only an existing damage, and its results are not always reproducible, whereas the micro-perimetry provides more reproducible and reliable results due to the eye-tracker function on the fixation. Furthermore, it provides information on fixation whose stability is a positive parameter after visual rehabilitation. Sensitivity was measured from 0 to 25 dB, and the color was coded. The field of the infrared image was 36° × 36°, and perimetry was performed in a field of 30° × 30° with a luminance of 4 asb. The full-threshold 4-L test was used to assess the retina in detail.

### LRRT: cell isolation and grafting procedures

The autograft of MCs in the suprachoroidal space, i.e., LRRT, consisted of the following triad: ASCs, ADSCs contained in SVF of adipose tissue, and platelets obtained from the PRP. ASCs were collected from the orbital fat during the surgical procedure according to previously published methods [8]. A scleral pocket with a deep sclerectomy was created in each patient’s eye to expose the surface of the choroidal space [9].

After exposing the choroid, the pedicle of adipose tissue derived from the orbital space was placed on the choroid’s surface (Fig. 1). ADSCs contained in the SVF were grafted in the suprachoroidal space. The SVF was isolated from the abdominal fat according to the Lawrence and Coleman technique [14]. Briefly, 10 mL of adipose tissue was manually harvested from the abdominal subcutaneous layer of each patient using a 3-mm blunt cannula connected to a locking syringe. After adding 50 mL of saline solution to the freshly harvested lipoaspirate for 10 min to eliminate the blood component, the supernatant was extracted and centrifuged at 1500×*g* for 5 min at 20°C in order to isolate SVF from the mature adipocytes, connective tissues, cellular debris, and oil.

The platelets were obtained from PRP gel according to the established methodologies [9]. Eight milliliters of human peripheral blood was collected with a 22-G needle and put in a Regen-BCT tube (RegenKit; RegenLab, Le Mont-sur-Lausanne, CH) for PRP preparation. The collected blood was centrifuged at 1500×*g* for 5 min at 20° C in order to isolate the PRP.

The adipose pedicle was infiltrated with platelets derived from the PRP gel (Fig. 1).

Finally, a mixture of ADSCs from the SVF and PRP was used to saturate the residual volume of the scleral pocket, where the pedicle of adipose tissue-derived from the orbital space was previously placed (Table 1).

**Table 1:** Surgical phases of the Limoli retinal restoration technique (LRRT)

• Anchoring of the sclera with 6-0 silk suture, near the inferior-temporal limbus, and globe deviated to the superonasal quadrant. • Opening of the sub-conjunctival and sub-Tenon’s space at 11 mm from the inferior-temporal limbus, using 5.5″ Westcott Tenotomy curved scissors. • Insert the Limoli-Basile conjunctival retractor in the space to make a scleral surgical field. • To pre-cut the flap on the side in the sclera at 8 mm from the limbus using a 5-mm crescent knife angled up with the flap hinge always radial and to the left of the surgeon. • Open a deep scleral flap of about 5 × 5 mm at the inferotemporal quadrant, maintaining the radial hinge. The sclerectomy has to be deep enough to allow viewing of the color of the choroid. • Remove a little operculum in the distal part of the flap in order to facilitate blood circulation in the subsequent suprachoroidal autograft. • Extract the orbital fat with forceps from a gap above the inferior oblique muscle. The fat must sufficiently be vascularized to allow it to survive after its implantation. • Place the autologous fat flap on the choroidal bed and suture with choroidal 6/0 polyglactin fiber at the proximal edge of the door. • Suture the scleral flap to avoid compression on the fat pedicle or its nutrient vessels. • Infiltrate the stroma of the fat pedicle with 1 mL of PRP gel (obtained by centrifugation of the blood material, separation of the component, and platelet degranulation) using a 30-G angled (30°) cannula. • Remove the conjunctival retractor. Suture the conjunctiva with 6/0 polyglactin fiber. • Leave a small flexible plastic tube to insert the autologous ADSCs in the space between the flap, the choroid, and the suprachoroidal autograft, before closing. • Fill the remaining space between the autologous fat graft, choroid, and scleral flaps with 0.5 cc of ADSCs in SVF and 0.5 of PRP using a 25-G cannula and close the suture. • After surgery, administer 3 days of antibiotic therapy with 500 mg of azithromycin. Also, provide eye drop therapy with an antibiotic and steroid combination, such as chloramphenicol and betamethasone, for about 15–20 days.	

### Cell identification by flow cytofluorimetry

Flow cytometry analyses were performed in order to identify the phenotypic characteristics of the population of cells within the graft, specifically ADSCs and platelets. PRP and SVF were obtained from patients of the LRRT group who underwent LRRT surgery and were isolated under fresh conditions. SVF was manually isolated from each patient’s lipoaspirate in a clean room near the operating room, according to a previously described method [15]. Briefly, the adipose portion of the lipoaspirate was washed with the phosphate-buffered saline (PBS; Biological Industries) and mixed with 2.5 mg/mL of collagenase type II (Worthington Biochemical Corporation, Lakewood, NJ) for enzymatic digestion. The collagenase/adipose mixture was placed in a 37°C water bath for 30 min in order to create a single-cell suspension and then filtered through a 100-μm cell strainer and finally a 45-μm mesh. It was centrifuged for 5 min at 1200×*g* to collect the cellular SVF as a pellet. Once isolated, characterization of the cell composition of freshly isolated SVF was obtained by multi-color flow cytometry (CytoFLEX Flow Cytometer, Backman Coulter, USA) that allows the in vitro identification of the surface marker expression of the cells. The panel of cell surface antigens was chosen in agreement with the International Federation for Adipose Therapeutics and Science (IFATS) and the International Society for Cellular Therapy (ISCT) recommendations [16]. The immunophenotypic analyses were performed to confirm the mesenchymal nature of isolated cells. The following fluorochrome-labeled monoclonal antibodies were used for SVF analysis: CD31-PE, CD34-PC, and CD45-APC (Backman Coulter, USA). The markers were used in combination with ViaKrome (Beckman Coulter, USA), which determines cell viability, excluding the debris and dead cells induced by the isolation protocol. Cells were incubated with specific mAbs for 15 min. At least 105 cells were acquired from each sample. The software CytEpert Version 2.2.0.97, CytoFLEX (Beckman Coulter, Inc.) was used to create dot plots and to calculate the cell composition percentages according to the profile of the surface marker expression. Immunophenotyping of platelets by flow cytometry was performed on platelets in PRP. The following markers were used for platelet analysis: anti-CD41-FITC and CD61-PE [17].

### Statistical analysis

All statistical analyses were performed using the software SPSS Statistics (version 20.0, SPSS Inc., Chicago, IL). Data were summarized with the mean ± standard deviation (SD), and minimum and maximum (min-max) values were also reported. Student’s *t* test was used to compare the values between controls and the study group. The paired *t* test was run to compare the study subjects and controls at baseline and after 6 months. A *p* value < 0.05 was considered statistically significant.

## Results

### Patient characteristics

A total of 35 eyes from 25 patients affected by GON (10 females and 15 males; mean age 70.7 ± 9.9 years, range 50–84 years) met the inclusion criteria and were enrolled in the study. Twenty-one eyes of the total composed the control group (8 males and 7 females; mean age 72.2 ± 9.6 years, range 50–83 years), while the remaining 14 eyes constituted the LRRT group of patients (7 males and 3 females; mean age 69.4 ± 9.2 years; range 50–84 years). Two patients from the latter group were excluded from the analysis because of the close-up visus that was not evaluable and the BCVA that was greater than 1 LogMAR. The baseline characteristics are summarized in Table 2.

**Table 2:** Characteristics of the Limoli retinal restoration technique (LRRT) group and control group

Parameters of patients	LRRT group	Controls
Number eyes (patients)	14 (10)	21 (15)
Age, years (range)	69.4 (50–83)	72.2 (50–84)
Sex, number (%), male	7 (70)	8 (54)
Sex, number (%), female	3 (30)	7 (46)

No adverse event associated with the surgery either intra-operatively or post-operatively was observed throughout the period. The mean values of the IOP recorded before and after surgery did not change significantly. In the control group, the mean IOP in T0 was 13.76 mmHg (range 10–15 ± 1.64 SD), and in T180, 13.33 mmHg (range 10–15 ± 1.39 SD). In the LRRT group, the mean IOP in T0 was 13.29 mmHg (range 10–15 ± 1.63 SD), and in T180, 13.29 mmHg (range 10–15 ± 1.59 SD). All completed 6 months of evaluation.

### Phenotype of platelets and freshly isolated SVF

The positive expression for cell surface antigens CD61 and CD41 identified PLTs. We observed that the PRP contained a mean of 79.2 ± 13.7% PLT on a total of 10^5^ cells.

**Fig. 2:**
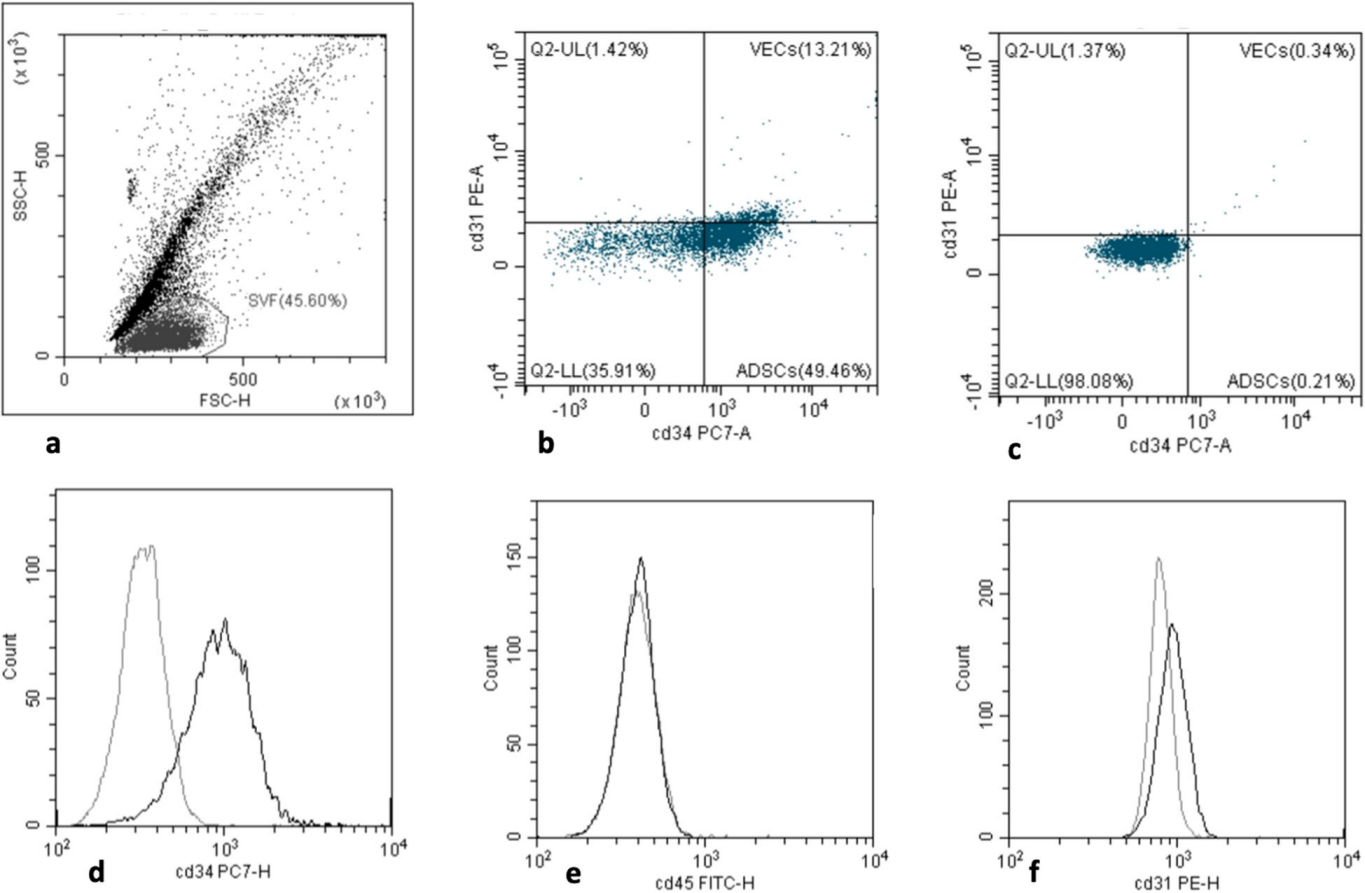
Flow cytometric histograms of adipose-derived stem cells (ADSCs) within the stromal vascular fraction (SVF) of a representative patient. Each histogram contains an isotype-matched negative Ab control (gray line) and Abs against the following Ags (black line). Representative fluorescent dot plot displaying cells within the freshly isolated lipoaspirate (**a**). Dot plot displaying mesenchymal stem cell (MSC) identified using antibodies directed against CD45, CD31, and CD34 (**b**) and negative control (**c**). Two-dimensional histograms displaying staining for CD45-FITC, the pan-hematopoietic marker (**d**); CD31-PE, found on endothelial cells, platelets, and leukocytes (**e**); and CD34-PC, a marker for pluripotent stem cells (**f**). Histogram of identical ADSC stained with anti-CD44 Abs conjugated to PE or FITC

The positive expression for cell surface antigens CD34 and negative expression for CD31 and CD45 identified ADSCs. The percentage of the phenotypically identified ADSC population was 44.9% ± 11% on a total of 10^5^ cells and the ADSC/μL was 590.3/μL (127.2–1485.3/μL) (Fig. 2).

### BCVA

**Fig. 3:**
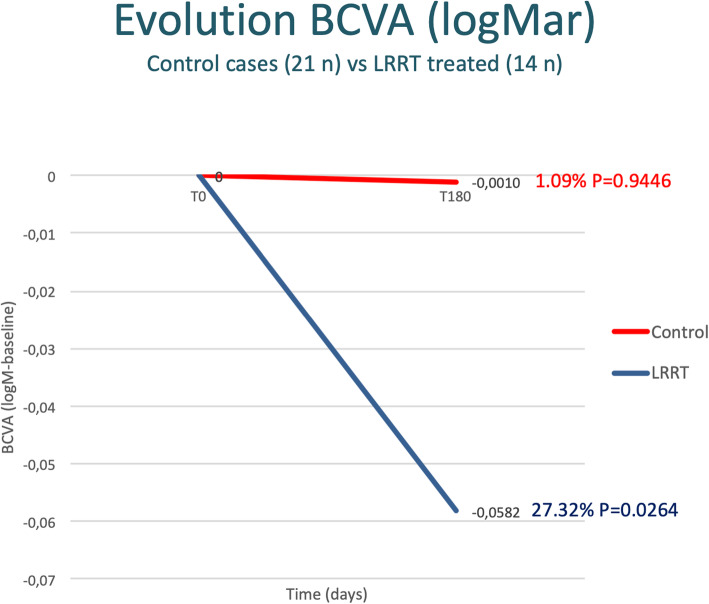
The autologous mesenchymal stem cell (MSC) graft supports the optic nerve promoting a significant increase in best-corrected visual acuity (BCVA) compared to the control group

After 6 months, the BCVA went from 0.0947 to 0.0937 logMAR in the control group with a mean increase of −0.001 (+ 1.09%; *p* > 0.05) and from 0.213 to 0.155 logMAR in the LRRT group with a statistically significant increase of −0.0582 (+ 27.32%; *p* = 0.0264) (Fig. 3; Table 3). The difference between the 6-month increase in the LRRT-treated group and the evolution in the control group is considered to be statistically significant (*p* = 0.0353).

**Table 3:** Best-corrected visual acuity (BCVA), close-up visus, and sensitivity in the Limoli retinal restoration technique (LRRT) and control groups. Values between the controls and LRRT groups were compared by Student’s *t* test at baseline (T0) and 6 months (T180)

	LRRT group (*N*=14)				Control (*N*=21)				*p*^b^
	Means ± SD		∆ ± SD	*p*^a^	Means ± SD		∆ ± SD	*p*^a^	
	T1	T180			T1	T180			
BCVA, LogMAR	0.213 ± 0.295	0.154 ± 0.246	−0.058 ± 0.087	0.0264*	0.095 ± 0.135	0.094 ± 0.104	−0.001 ± 0.067	0.9446	0.0353*
%			27.3%				1.1%		
Close-up visus, pts	10.21 ± 7.44	8.29 ± 5.59	1.93 ± 5.37	0.2009	6.57 ± 1.47	6.90 ± 1.92	−0.33 ± 1.80	0.4057	0.0818
%			18.8%				−5.02%		
Sensibility, dB	10.00 ± 5.82	11.12 ± 6.00	1.12 ± 1.17	0.0033*	13.20 ± 5.90	12.64 ± 5.83	−0.56 ± 1.53	0.1093	0.0014*
%			11.2%				−4.2%		

*SD* Standard Deviation, *logMAR* logarithm of the minimum angle of resolution, *pts* points, *dB* decibel, *∆* mean changes from T0 to T180

*Statistical significance with *p* value ≤ 0.05

^a^Intergroup comparison

^b^Comparison between the groups

### Close-up visus

**Fig. 4:**
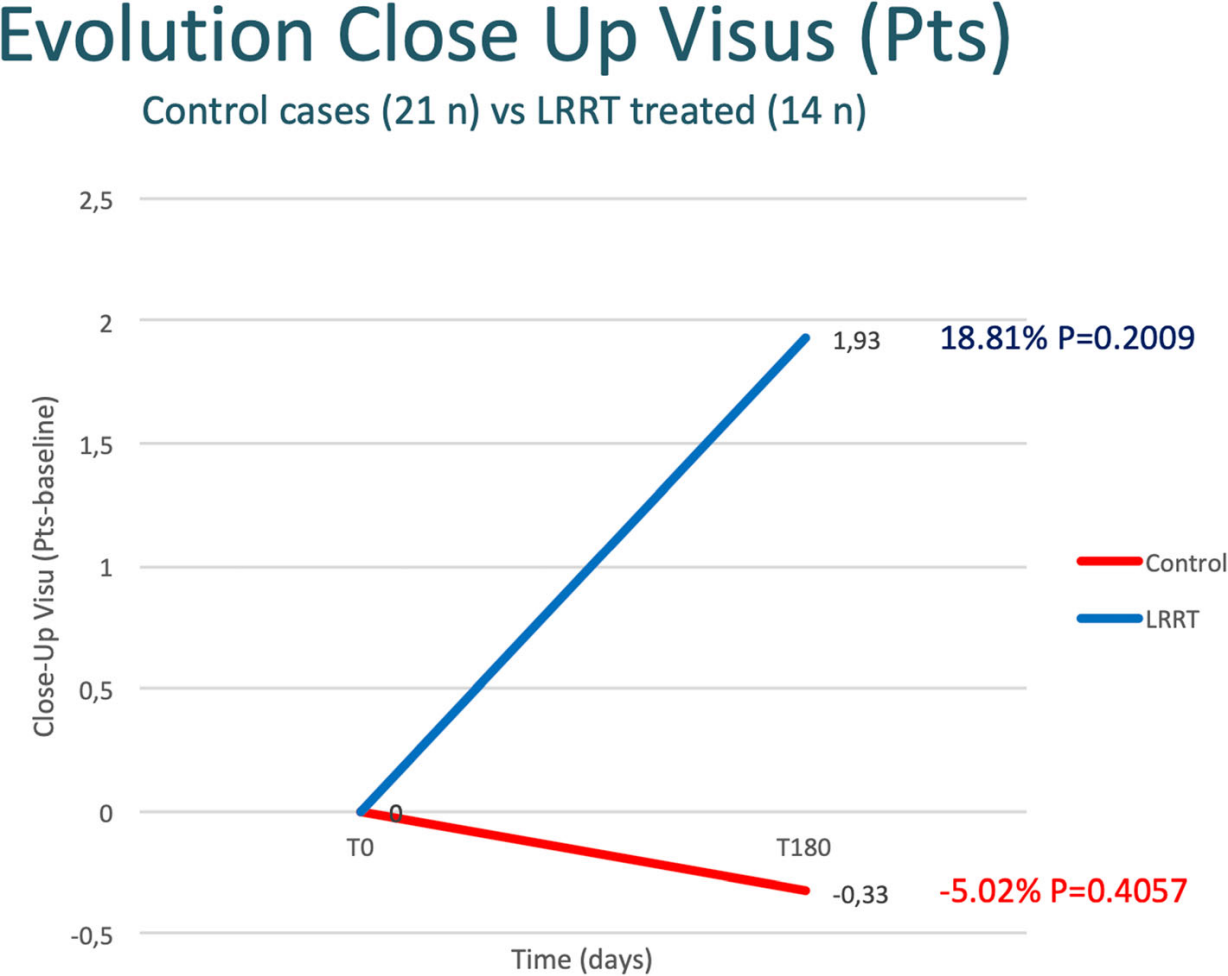
The autologous mesenchymal stem cell (MSC) graft supports the optic nerve promoting an increase in close-up visus compared to the control group, but not in a statistically significant manner

After 6 months, the close-up visus went from 6.57 to 6.9 pts in the control group with a mean reduction of −0.33 pts (−5.02%; *p* >0.05) and from 10.21 to 8.29 pts in the LRRT-treated group with a mean increase of 1.93 (+18.81%; *p* >0.05) (Fig. 4, Table 3). However, the latter increase did not reach statistical significance. The difference between the 6-month increase in the LRRT-treated group compared to the evolution in the control group is not considered to be statistically significant (*p* = 0.0818).

### Microperimetry

**Fig. 5:**
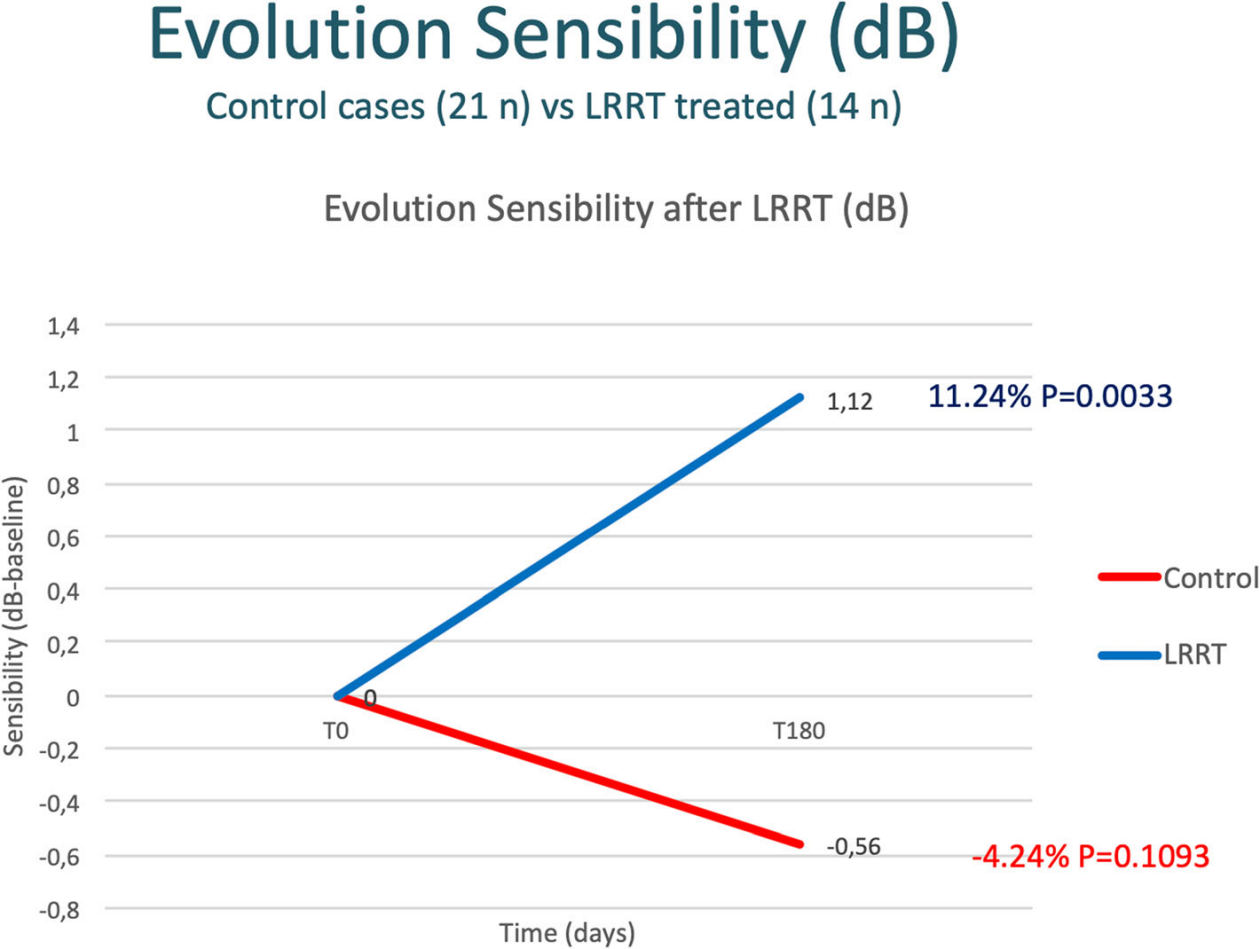
The autologous mesenchymal stem cell (MSC) graft supports the ocular sensitivity promoting a significant increase in sensibility compared to the control group

**Fig. 6:**
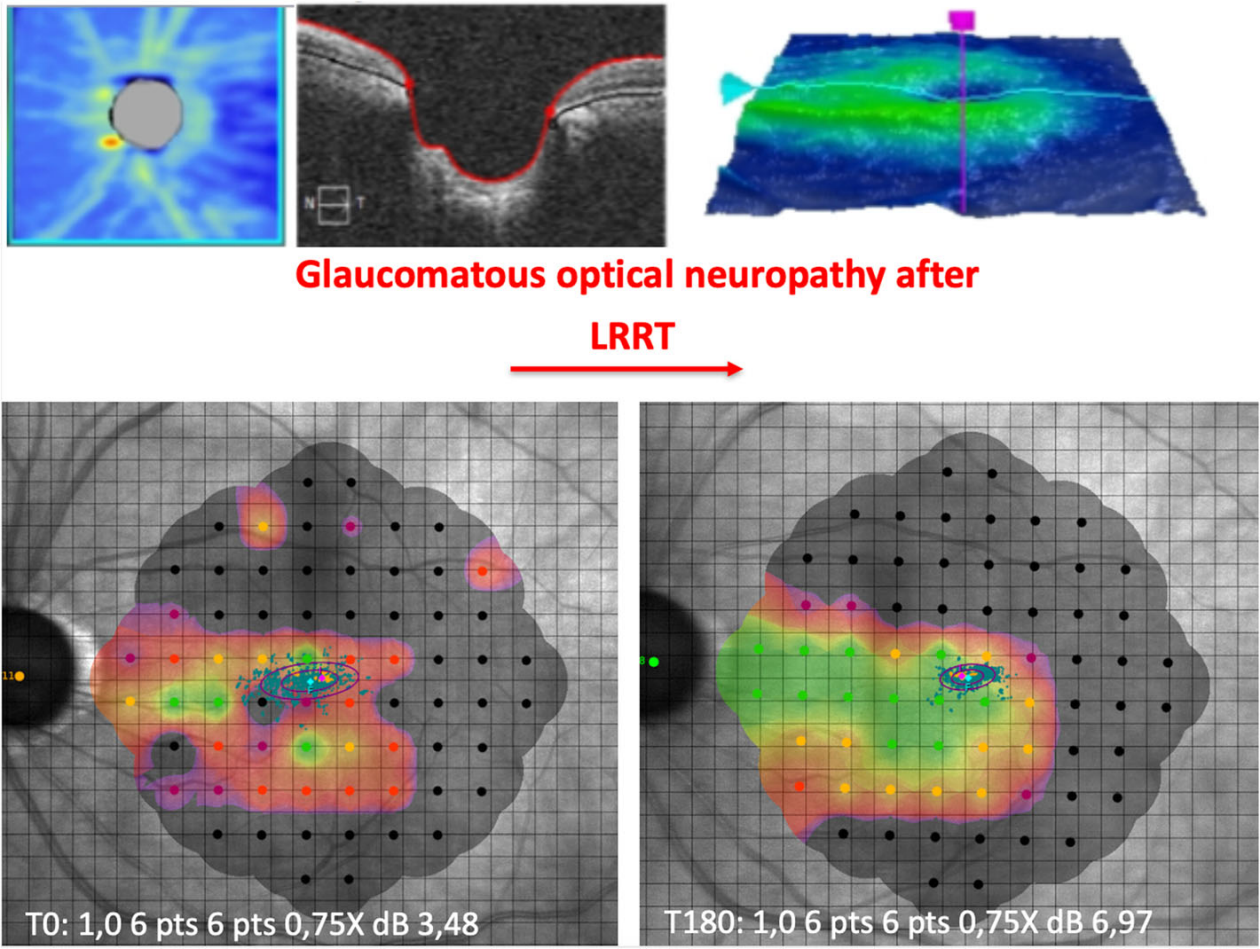
Microperimetry of a patient before and after surgical treatment. After 6 months, the sensitivity has improved

After 6 months, sensitivity went from 13.20 to 12.64 dB in the control group with a mean reduction of −0.56 dB (−4.24%; *p* > 0.05) and from 10.00 to 11.12 in the LRRT-treated group with a statistically significant increase of 1.12 dB (+11.24%; *p* = 0.0033) (Figs. 5 and 6, Table 3). The difference between the 6-month increase in the LRRT-treated group compared to the evolution in the control group is considered to be statistically significant (*p* = 0.0014).

### Compliance

**Fig. 7:**
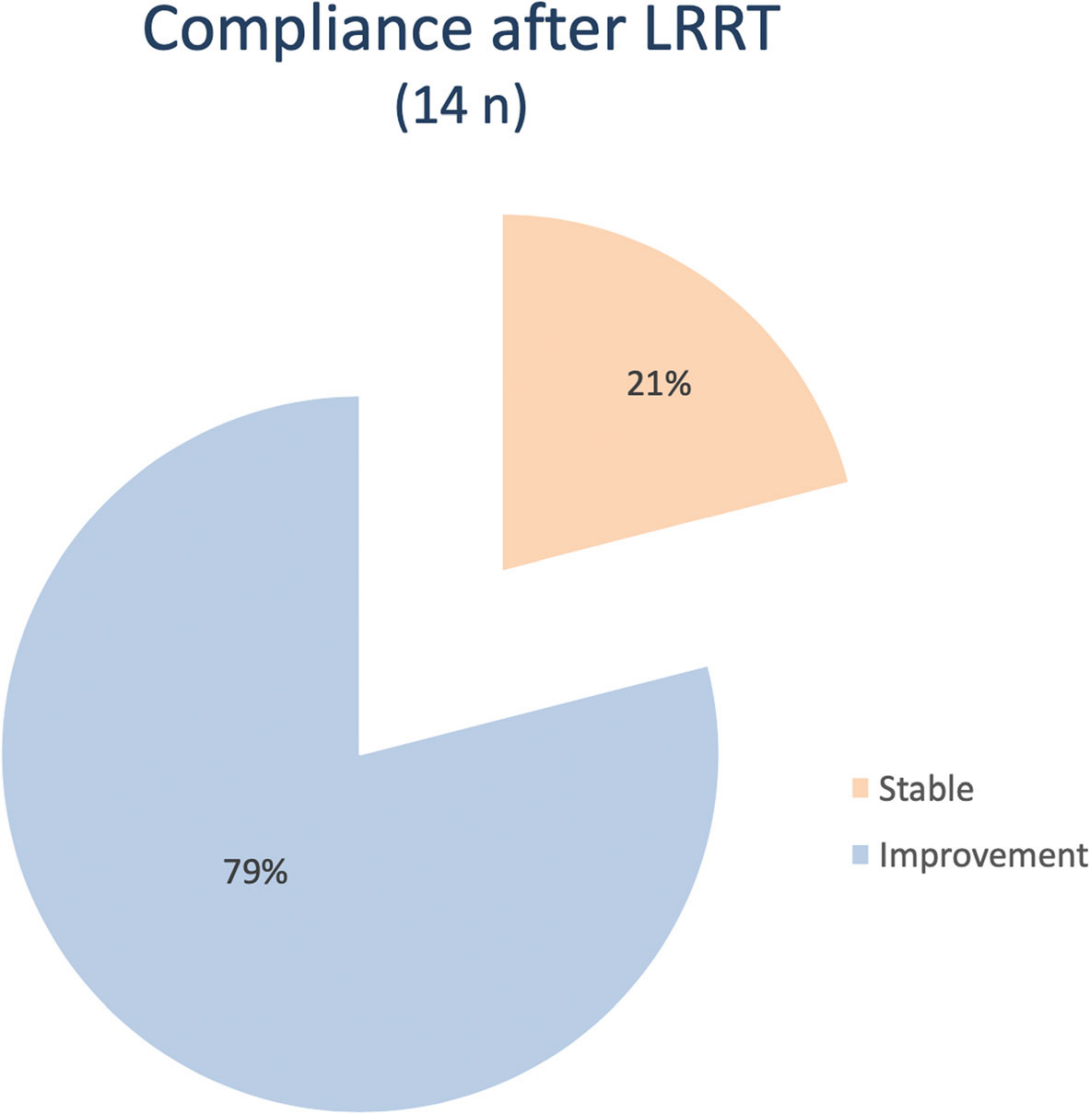
No patient reported seeing worse after the implant or experiencing ocular complications. Instead, 80% of the treated patients reported a subjective functional improvement

The subjective experience of all LRRT-treated patients was surveyed. At 6 months, the VP increased in 11 eyes out of 14 (79%) and remained unvaried in 3 eyes (21%). Notably, the VP worsened in no eyes (Fig. 7).

## Discussion

In this study, patients affected with GON received the LRRT treatment to preserve the residual VP. All of the eyes in the study group showed improvement in BVCA, sensitivity, and residual close-up visus with no ocular and systemic complications, whereas the majority of the eyes in the control group showed a decrease in the same measured parameters. The improvements were consistent through the 6-month follow-up. Furthermore, we monitored IOP throughout the study and included patients affected with GON whose IOP was well within normal parameters. There were no variations in our case study.

GON is currently recognized as a progressive neurodegenerative disease, resulting in due course permanent visual loss [2]. Up to date, there are no curative treatments; however, many potential options are being investigated in the clinical setting, including retinal prostheses, gene therapies, and cell-based treatments. Among these different therapeutic alternatives, growing research interest has been developed towards the MSCs, i.e., adult stromal cells, as promising candidates for cell therapy in retinopathies [6–8, 18].

These cells are ubiquitously distributed in the body and play a key role in organogenesis, tissue remodeling, and repair [18]. A growing body of evidence points that MSCs can restore VP in different ocular degenerative disorders through various therapeutic pathways involving cell differentiation to replace the lost cells, paracrine activity to trigger cell survival and repair, and modulation of the local immune response [6–8, 18].

They are characterized by multipotency, as they have been shown to differentiate into several cell types, including adipocytes, chondrocytes, osteoblasts, vascular endothelial cells, cardiomyocytes, beta-pancreatic cells, hepatocytes, and, under specific conditions, also into retinal neuron-like, including retinal progenitors and photoreceptors [19]. However, MSCs are clinically attractive for the paracrine secretion of a broad range of bioactive molecules such as cytokines, chemokines, and GFs [20–22]. To name a few, they can secrete basic fibroblast growth factor (bFGF), vascular endothelial growth factor (VEGF), macrophage colony-stimulating factor (M-CSF), granulocyte-macrophage colony-stimulating factor (GM-CSF), placental growth factor (PlGF), transforming growth factor-β (TGF-β), hepatocyte growth factor (HGF), insulin-like growth factor-1 (IGF-1), interleukin (IL), angiogenin, ciliary neurotrophic factor (CNTF), brain-derived neurotrophic factor (BDNF), and glial cell-derived neurotrophic factor (GDNF) [18, 23]. Also, MSCs can release extracellular vesicles and exosomes that carry mRNA, microRNA, proteins, mitochondrial components, and ribosomes [24]. It is known that exosomes help communication between MSCs and the surrounding niches and can activate the proliferation and differentiation of native stem cells [24].

These biological mediators are well known for providing a vital microenvironment by inducing gene expression changes that lead to neuroprotective, regenerative, anti-inflammatory, and anti-apoptotic effects; hence, they can support cell survival and rescue the damaged tissue [6–8, 18]. This complex interplay has been shown to cause functional neuroenhancement of the residual retinal cells and to regulate regeneration by reversing cell death or damage in different diseases. Especially due to their paracrine trophic activity, MSCs have emerged as trending regenerative biologic agents for retinopathies [18].

Moreover, growth factors secreted by the grafted cells in cell therapy could influence the autophagic processes. Autophagic markers increase in optic nerve damage conditions such as glaucoma. In fact, ultrastructural and biochemical results have shown that autophagy is significantly activated in RGCs after chronic IOP elevation. Then, it reduces after a few weeks [25]. The use of stem cells could modulate autophagy through the secretion of TGFβ in the cell medium which induces an increase of α-smooth muscle actin (αSMA) levels, necessary for autophagy activation [26]. During the autophagic process, organelles and unwanted or damaged molecules are degraded by lysosomes, thus leading to the formation of nucleotides, amino acids, and free fatty acids that can be reused for the synthesis of essential macromolecules and for energy production [26]. In this way, the cell can recycle waste material in order to increase its survival [27].

As defined by the International Society for Cellular Therapy, MSCs are characterized by a set of negative and positive surface markers; they express CD105, CD73, CD34, and CD90 and lack CD45, CD14, CD11, CD79, CD19, and HLA-DR. MSCs also express other surface markers, such as CD44, CD166, Stro-1, CD106, and CD146 [16].

MSCs can be isolated from adult and fetal tissues, including bone marrow, adipose tissue, Wharton’s jelly, dental pulp, and placenta [20, 28]. ADSCs have been emerging as ideal MSCs among the other cell sources because of their sustainable costs, manageability, easy harvesting, and wide distribution in the adult tissues. Compared with bone marrow, adipose tissue contains a higher number of MSCs and of pericytes, which are the precursors to MSCs, and a lower amount of leukocytes [20, 28]. Furthermore, the adipose tissue is one of the most attractive sources for MSCs due to the lack of ethical concerns involved in their application, and the high paracrine trophic and immunomodulatory effects. Most notably, ADSCs have been shown to have no risks of uncontrolled growth and malignant transformation and no rejection or immune reactions, demonstrating their long-term efficacy and compatibility in the transplanted tissue [22, 28]. For all the provided reasons, ADSCs are ideal for autologous cell transplants, and we chose to use them for our surgical procedure.

The LRRT is a cell therapy consisting of autologous ADSCs within the SVF, ASCs, and PRP [9]; it is administered intra-ocularly with a supra-choroidal delivery method.

Alongside ADSCs, also ASCs have shown regenerative potential as well as autologous PRP that is a source of growth factors [8, 28]. Freshly isolated cells were subjected to flow cytometry analyses to confirm the immunophenotyping characterization of ADSCs within SVF and PRP.

We want to remind that some ways different of administration of MSCs have been explored in several clinical studies for the management of degenerative retinal diseases. We used the suprachoroidal method, which is reported to have no serious complications and is considered to be safer compared to the intravitreal or subretinal applications [11].

The choice to use the suprachoroidal route, rather than other forms of administration, is determined by clinical safety as well as by good efficacy. The sub-tenonian route has been used with discrete efficacy and absolute safety to graft stem cells [29] and PRP [30] in the course of retinitis pigmentosa. Other routes have been used for the same purpose such as the intravitreal route [31] and the subretinal route [32], but several and sometimes severe side effects have been reported. The suprachoroidal, used by other researchers, route consists of placing the material externally at the choroid and has shown to be very safe and effective [33, 34]. To the best of our knowledge, there are not any studies using the sub-tenonian route for mesenchymal cell graft in either opticopathy or optic atrophy. The present work aims to highlight the behavior of visual performance in patients who receive cell treatment compared to those who do not. The suprachoroidal technique has not shown any damage and did not have any adverse side effects in any case. In the future, the efficacy and safety of the sub-tenonian route in comparison with the suprachoroidal one could be evaluated.

The suprachoroidal area has been shown as a natural drug storage and an immune-protected region [9–13]. The involved supra-choroidal area is only 25 mm^2^, thus representing a minimal space of the whole bulbar surface. Also, in LRRT surgery, there is no compression of the supra-choroidal space as the scleral flap under which is situated the cell graft is gently sewn. This is very important because if the cell graft is compressed, the adipose pedicle inserted could become ischemic and atrophic. The GFs secreted by the ADSCs can effectively pass through that space and reach the retinal target without producing immune reactions, making the suprachoroidal region ideal as the site of MSC administration.

The effect of ADSCs is thought to be related to the expression of several GFs, including bFGF, BDNF, NGF, CNTF, GDNF, and HGF [3, 6, 7]. GFs secreted by MSCs in the suprachoroidal space can either trigger the retinal cells in the quiescent phase to re-enter the cell cycle and activate the progenitor cells or act directly on the damaged cells supplying neuroprotection and reducing the retinal oxidative damage. GFs have been shown to inhibit apoptosis in the diseased retina, to mediate a neurocytoprotective action, and to suppress retinal chronic inflammation that occurs in glaucoma through anti-inflammatory and immunomodulating action. Furthermore, several in vivo and in vitro studies have shown MSC-mediated pleiotropic activity in stimulating angiogenesis in ischemic disease, myelination, dendritic and axonal regeneration through IGF secretion, and mTOR pathway activation [20–38].

In this way, it can promote RGC survival and stimulate both axonal regeneration and myelination in the optic nerve, restoring both dendritic and synaptic connections with bipolar and amacrine cells [23–36].

According to these findings, MSCs might promote RGC function and survival through the paracrine release of GFs, exosomes, and microvesicles over time, slowing retinal degeneration.

These biochemical mechanisms could underlie the positive clinical results we observed following the autologous MSC graft performed by LRRT treatment in the suprachoroidal space in patients affected with GON.

The LRRT treatment has been applied in other studies of our group in retinal diseases, such as retinitis pigmentosa, AMD, and optic neuropathies, and its safety and effectiveness with improvements of both VP and electroretinographic parameters has been shown [9–12]. In this clinical study, the LRRT demonstrated healing potential in patients with GON.

In accordance with our results, many investigators evaluated the safety and efficacy of the MSC use for retinal diseases and suggest MSC-mediated neuroprotection [13, 29, 39, 40].

Oner et al. [13] showed that the suprachoroidal implantation of ADSCs in patients with optic nerve disease caused functional improvement in VP in terms of visual acuity, visual field, and mfERG recordings. These outcomes are believed to be related to the paracrine secretion of neurotrophic and angiotrophic GFs from ADMSCs and angiotrophic GFs from PRP, suppressing the inflammation and protecting RGCs from death.

In Brazil, Siqueira et al. [40] conducted a study with intravitreal injection of bone marrow-derived stem cells in patients with RP, showing the safety of the cell therapy and observing an increased quality of life.

Finally, a Californian group [41] obtained similar results with the intravitreous use of BMDSCs in patients affected with retinal vascular occlusion, non-exudative age-related macular degeneration, or retinitis pigmentosa. The investigators assessed the safety and feasibility of cell therapy, showing the important role that MSCs may play in tissue repair. The study has some limitations. First, our sample size was small, and the study was not masked. A larger number of patients will be necessary to evaluate the effects of this therapy.

Second, the duration of action of LRRT treatment is unknown. Even though long-term research is necessary to determine the duration of efficacy, PRP booster injections after 12 months have been shown to maintain the outcomes. Another limitation of the study is that we do not measure whether additional treatments such as electrical stimulation may increase MSC activity. The latter limitation forms the basis for near-future studies.

## Conclusions

The LRRT surgery has proven safe and effective in treating patients affected with GON.

In our experience, both visual acuity and retinal sensitivity measurements showed statistically significant improvements in 80% of GON patients after LRRT during the follow-up period of 6 months, and no ocular or systemic effects were reported. Therefore, autologous MSC graft combined with PRP into the suprachoroidal space could contribute to restoring optic nerve function, improving the clinical, prognostic, and rehabilitative aspects in patients affected with GON that currently have no curative treatment options.

Further studies are needed to validate our findings and to unveil the potential of MSCs as therapeutic agents in regenerative medicine especially for degenerative retinal and optic nerve diseases.

## Data Availability

The data that support the findings of this study are available from the corresponding author upon reasonable request.

## Abbreviations

ADSCs: Adipose-derived stem cells
AMD: Age-related macular diseases
ASCs: Adipose stromal cells
BCVA: Best corrected visual acuity
BDNF: Brain-derived neurotrophic factor
bFGF: Basic fibroblast growth factor
CNTF: Ciliary neurotrophic factor
ESC: Embryonic stem cell
ETDRS: Early treatment diabetic retinopathy study charts
GDNF: Glial cell-derived neurotrophic factor
GFs: Growth factors
GM-CSF: Granulocyte-macrophage colony-stimulating factor
GON: Glaucomatous optic neuropathy
HGF: Hepatocyte growth factor HIF-1
HIF-1: Hypoxia-inducible factor 1
IGF: Insulin-like growth factor
IOP: Intraocular pressure
iPSC: Induced pluripotent stem cell
logMAR: Logarithm of the minimum angle of resolution
LRRT: Limoli retinal restoration technique
M-CSF: Macrophage colony-stimulating factor
MSC: Mesenchymal stem cell
NGF: Nerve growth factor
PLTs: Platelets
PRP: Platelet-rich plasma
pts: Points or print size
RGC: Retinal ganglion cell
RNA: Ribonucleic acid
ROS: Reactive oxygen species
RP: Retinitis pigmentosa
RPE: Retinal pigment epithelium
SD-OCT: Spectral domain-optical coherence tomography
SVF: Stromal vascular fraction
TGF-β: Transforming growth factor-β
TNF-α: Tumor necrosis factor-α
VEGF: Vascular endothelial growth factor
VP: Visual performances
